# Supplementary material for: MetaRibo-Seq measures translation in microbiomes
Source: Nat Commun. 2020 Jun 29;11:3268. doi: 10.1038/s41467-020-17081-z (PMC7324362; doi:10.1038/s41467-020-17081-z)
Supplement: Supplementary file 10 — Supplementary Data 7 [file 41467_2020_17081_MOESM10_ESM.zip › File2/Confidence_High_Taxonomy/333004_out.krona.html]

Javascript must be enabled to view this page.

members
magnitude
magnitudeUnassigned
count
unassigned
taxon
rank

333004\_out

65

2
superkingdom
65

phylum
65
976

class
65
200643

171549
order
65

171552
family
64

genus
64
838


SRS011134\_contig\_number\_26008SRS012849\_contig\_number\_14370SRS012969\_contig\_number\_13162SRS013521\_contig\_number\_3810SRS013638\_contig\_number\_2024SRS013687\_contig\_number\_18811SRS014415\_contig\_number\_1832SRS015794\_contig\_number\_2698SRS016541\_contig\_number\_15259SRS017307\_contig\_number\_18196SRS018888\_contig\_number\_2445SRS019910\_contig\_number\_13713SRS022609\_contig\_number\_27494SRS023526\_contig\_number\_37088SRS023715\_contig\_number\_contig-100\_56.120860SRS023914\_contig\_number\_27661SRS024132\_contig\_number\_23542SRS043667\_contig\_number\_744SRS043841\_contig\_number\_10659SRS044535\_contig\_number\_12164SRS045713\_contig\_number\_22313SRS046502\_contig\_number\_4373SRS047433\_contig\_number\_24641SRS049712\_contig\_number\_10814SRS049995\_contig\_number\_14245SRS053356\_contig\_number\_47696SRS053398\_contig\_number\_19946SRS054059\_contig\_number\_2539SRS058145\_contig\_number\_333SRS064757\_contig\_number\_17732SRS065397\_contig\_number\_contig-100\_199.96631SRS074586\_contig\_number\_contig-100\_107.40880SRS077194\_contig\_number\_12940SRS077641\_contig\_number\_1416SRS077849\_contig\_number\_12654SRS078176\_contig\_number\_2864SRS078419\_contig\_number\_25999SRS097958\_contig\_number\_1955SRS104036\_contig\_number\_6478SRS1041031\_contig\_number\_30180SRS1041134\_contig\_number\_13411SRS1041137\_contig\_number\_29333SRS1041140\_contig\_number\_10526SRS104327\_contig\_number\_4361SRS105082\_contig\_number\_3430SRS143181\_contig\_number\_11171SRS143876\_contig\_number\_5455SRS144297\_contig\_number\_contig-100\_520.90929SRS144506\_contig\_number\_35481SRS144537\_contig\_number\_18990SRS146764\_contig\_number\_15544SRS148253\_contig\_number\_12862
165179
species
52


SRS012989\_contig\_number\_2903SRS013098\_contig\_number\_12372SRS015782\_contig\_number\_14711SRS047044\_contig\_number\_2218SRS050752\_contig\_number\_20720SRS077991\_contig\_number\_contig-100\_84.23991SRS103987\_contig\_number\_553SRS147614\_contig\_number\_9481SRS150029\_contig\_number\_16634
species
9
59823


SRS049959\_contig\_number\_12308
2293125
1
species

159272
2
species

SRS017375\_contig\_number\_contig-100\_123.18128SRS019285\_contig\_number\_contig-100\_44.21934

family
1
815

816
1
genus


SRS144603\_contig\_number\_1956
1
species
821
